# Single dose of DPX-rPA, an enhanced-delivery anthrax vaccine formulation, protects against a lethal *Bacillusanthracis* spore inhalation challenge

**DOI:** 10.1038/s41541-019-0102-z

**Published:** 2019-02-08

**Authors:** Genevieve M. Weir, Lisa D. MacDonald, Rajkannan Rajagopalan, Gloria S. Sivko, Michelle W. Valderas, Jonathan Rayner, Bradley J. Berger, Leeladhar Sammatur, Marianne M. Stanford

**Affiliations:** 1 IMV Inc., 130 Eileen Stubbs Avenue, Suite 19, Dartmouth, NS B3B 2C4 Canada; 2 Battelle, 1425 Plain City Georgesville Road, West Jefferson, OH 43162 USA; 3 Southern Research, 2000 9th Avenue S, Birmingham, AL 35205 USA; 4 Suffield Research Centre, Defence Research and Development Canada, Medicine Hat, AB T1A 8K6 Canada; 5 Department of Microbiology and Immunology, Dalhousie University, 5850 College Street, Room 7-C, PO BOX 15000, Halifax, NS B3H 4R2 Canada

## Abstract

Anthrax is a serious biological threat caused by pulmonary exposure to aerosolized spores of *Bacillus anthracis*. Biothrax^®^ (anthrax vaccine adsorbed (AVA)) is the only Food and Drug Administration-licensed vaccine and requires five administrations over 12 months with annual boosting to maintain pre-exposure prophylaxis. Here we report the evaluation of a single intramuscular injection of recombinant *B. anthracis*-protective antigen (rPA) formulated in the DPX delivery platform. Immune responses were compared to an alum-based formulation in mice and rabbits. Serological analysis of anti-rPA immunoglobulin G and toxin neutralization activity demonstrated higher responses induced by DPX-rPA when compared to rPA in alum. DPX-rPA was compared to AVA in rabbits and non-human primates (NHPs). In both species, DPX-rPA generated responses after a single immunization, whereas AVA required two immunizations. In rabbits, single injection of DPX-rPA or two injections of AVA conferred 100% protection from anthrax challenge. In NHPs, single-dose DPX-rPA was 100% protective against challenge, whereas one animal in the two-dose AVA group and all saline administered animals succumbed to infection. DPX-rPA was minimally reactogenic in all species tested. These data indicate that DPX-rPA may offer improvement over AVA by reducing the doses needed for protective immune responses and is a promising candidate as a new-generation anthrax vaccine.

## Anthrax: Rapid immunity to a biological threat

A lipid-based anthrax vaccine formulation offers immunity from the first injection. *Bacillus anthracis* is a lethal pathogen at high risk for use in biological warfare. The only FDA-licensed vaccine for anthrax, AVA, requires multiple doses over six months followed by regular boosters, indicating a need for rapidly immunizing vaccines. Genevieve Weir and Lisa MacDonald, from IMV Inc., with Canadian and US collaborators, here describe a prophylactic consisting of *B. anthracis* antigens suspended in a lipid-in-oil formulation. Their candidate, DPX-rPA, generated antigen-specific antibodies in rabbits and monkeys after one dose, compared to two for AVA. DPX-rPA also protected both species from *B. anthracis* spores after one dose. The results indicate that single-dose DPX-rPA is equally protective as two doses of AVA and could serve as pre-exposure and post-exposure prophylaxis. Future studies may confirm its potential as a vaccine for humans.

## Introduction

Anthrax is a rapid-onset disease considered a serious biological warfare threat. Anthrax is caused by *Bacillus anthracis* (*B. anthracis*), a spore-forming Gram-positive bacterium that can be transmitted by gastrointestinal, cutaneous, or inhalation exposure routes. Pulmonary exposure to anthrax spores is typically the most lethal route of infection and is characterized by initial flu-like symptoms followed by fulminant respiratory distress with dyspnea, stridor, cyanosis, and chest pain. The onset of respiratory distress is associated with nearly 100% mortality if untreated.^1–4^

The current vaccine for anthrax, anthrax vaccine adsorbed (AVA; BioThrax^®^), is approved for pre-exposure and post-exposure prophylaxis of disease in person at high-risk of exposure and for persons with suspected or confirmed exposure in conjunction with antibiotics, respectively. AVA consists of a culture filtrate from an attenuated strain of *B. anthracis* adsorbed to aluminum salts as an adjuvant. The predominant means of protection provided by AVA is thought to be mediated by antibodies generated against protective antigen (PA), interfering with PA-mediated cellular entry of the anthrax toxins lethal factor (LF) and edema factor (EF).^5^ Product specification for AVA requires 5 to 20 μg/mL of total protein, of which at least 35% is the 83-kDa PA protein. While AVA is considered safe and efficacious, the pre-exposure vaccination regimen consists of three priming doses at 0, 1, and 6 months, with booster doses given at 6 and 12 months after completion of the priming series and annually thereafter. This regimen is not favorable to immunization of the general population and may contribute to injection site issues such as subcutaneous nodules, erythema, and induration.^6^ Additionally, although recently approved for a post-exposure setting, the US Centers for Disease Control and Prevention (CDC) recommends at least 5 weeks of continuous antibiotic treatment in parallel with vaccination, which is also challenging for widespread deployment. Anti-toxins are also available to complement antibiotic treatment; however, these may be expensive and have logistical considerations for widespread deployment.^7^ Due to concerns with the protracted dosing regimen and reactogenicity of AVA, alternative vaccine options that require fewer injections to promote rapid, enhanced protective immunity are currently under investigation for both pre-exposure and post-exposure prophylaxis measures.

As PA is believed to be the functional immunogen in AVA, alternative vaccine approaches have focused on approaches using recombinant PA (rPA) as a single antigen. This subunit approach offers the advantages of a synthetic, characterizable product that can induce immune responses to a single immunogen.^8^ This approach may also offer an improved safety profile compared to AVA, which is prepared using culture supernatants. To boost immune responses towards rPA, vaccines are usually prepared with an alum adjuvant. Although these vaccines have demonstrated the ability to induce protective antibody responses, their efficacy can wane over time due to stability issues in storing alum-based vaccines.^9,10^

The current study investigated the immunogenicity and protective response of rPA antigen formulated with the DPX^TM^ no-release delivery platform (DPX-rPA). DPX is a patented formulation that provides controlled and prolonged delivery of antigens and adjuvant to the immune system. The platform is composed of lipid-mixture nanoparticles admixed with adjuvant and antigen, lyophilized, and then suspended in mineral oil for solubilization. This unique formulation promotes a depot effect that attracts antigen-presenting cells to the vaccination site and elicits an immune response following single-dose delivery. DPX does not require creation of an emulsion, simplifying its use as an oil-based vaccine, and can be stored in lyophilized form, maintaining stability of the antigen. Preclinical testing has demonstrated that a single dose of antigen formulated in DPX can confer stronger and longer lasting immune responses towards peptide or protein antigens compared to multiple doses of alum-based formulations with the same antigen.^11–13^ The DPX platform can be formulated with different antigens and adjuvants to tailor responses towards different indications.^11,12^ A vaccine candidate for respiratory syncytial virus (RSV), DPX-RSV(A), was developed using the small hydrophobic antigen from RSV and Pam3CSK4 adjuvant and evaluated in healthy adults (50–64 years). DPX-RSV(A) induced antigen-specific antibodies after two immunizations, 56 days apart, which were sustained for at least 180 days, and over a year in the high-dose cohort.^14^ DPX has also been formulated with cancer antigens and tested in various indications in clinical trials.^15,16^ The product DPX-Survivac, which contains multiple major histocompatibility complex class I antigens derived from the survivin protein, is in phase 2 clinical evaluation.

In this study, we investigated the immunogenic potential of DPX-rPA compared with PA antigen admixed with alum or AVA in mice, rabbits, and non-human primates (NHPs). The results demonstrate the ability of DPX-rPA to generate functional anti-rPA immunoglobulin G (IgG) in serum and to confer protection from aerosolized lethal *B. anthracis* spore challenge in multiple species.

## Results

### Single-dose delivery of DPX-rPA in mice elicits rapid and sustained anti-rPA IgG response

Preliminary studies were performed using outbred CD-1 mice to optimize the DPX-rPA formulation. We compared responses to the same antigen formulated in alum, as alum has been used by others to induce antibody responses to rPA.^8^ CD-1 outbred mice were used as they typically generate robust antibody responses to a wide variety of antigen candidates and can better represent diversities in immune background when compared to inbred mice. Mice were vaccinated intramuscularly with a single dose of either 10, 4, 2, 0.5, or 0.05 µg of rPA in DPX (DPX-rPA) or with 10 µg of rPA in alum (alum-rPA). Serum anti-rPA IgG titers were measured in vitro from samples harvested at Weeks 3, 4, 8, 12, 16, and 20 post-immunization using an enzyme-linked immunosorbent assay (ELISA).

**Fig. 1:**
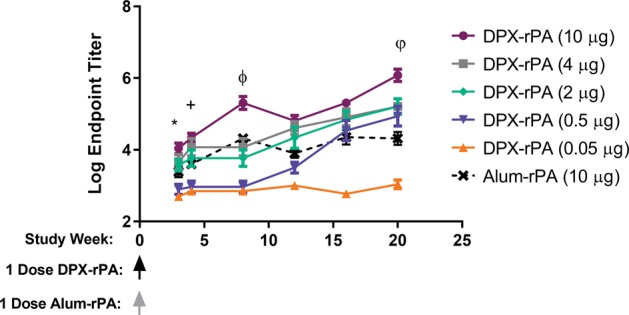
Single-dose delivery of DPX-rPA (recombinant protective antigen) in mice induces rapid and sustained anti-rPA immunoglobulin G (IgG) response. Groups of nine CD-1 mice were vaccinated (intramuscularly (i.m.)) once with DPX-rPA dosed at either 10 µg (purple circles), 4 µg (gray squares), 2 µg (green diamonds), 0.5 µg (dark purple downwards triangles), or 0.05 µg (orange upwards triangles) or once with 10 µg alum-rPA control vaccine (black x). Immunizations are indicated with arrows. Endpoint anti-rPA IgG titers, presented as Log_10_ values, at Weeks 3, 4, 8, 12, 16, and 20 post immunization determined by enzyme-linked immunosorbent assay (ELISA) using plates coated with rPA antigen. Results are shown as mean ± SEM. Significance shown was determined by two-way analysis of variance (ANOVA) with Tukey’s multiple comparisons test of at least *p* < 0.05, on Weeks 3, 4, 8 and 20: *, DPX-rPA (0.05 μg) compared to DPX-rPA (2, 4, 10 μg), DPX-rPA (0.5 μg) vs. DPX-rPA (4, 10 μg); +, DPX-rPA (0.05 μg) vs. DPX-rPA (2, 4, 10 μg), DPX-rPA (0.5 μg) vs. DPX-rPA (2, 4, 10 μg); ϕ, DPX-rPA (0.05 μg) vs. DPX-rPA (2, 4, 10 μg), and alum, DPX-rPA (0.5 μg) vs. DPX-rPA (2, 4, 10 μg) and alum, DPX-rPA (2 μg) vs. DPX-rPA (10 μg), DPX-rPA (4 μg) vs. DPX-rPA (10 μg), DPX-rPA (10 μg) vs. alum; φ, DPX-rPA (0.05 μg) vs. all other groups, DPX-rPA (0.5 μg) vs. DPX-rPA (μg), DPX-rPA (2 μg) vs. DPX-rPA (10 μg), DPX-rPA (4 μg) vs. DPX-rPA (10 μg), DPX-rPA (10 μg) vs. alum

DPX-rPA elicited a rapid and sustained antibody response directed against PA that corresponded with increasing antigen dose, initiating within 3 weeks of delivery, and persisting in serum for up to 20 weeks (Fig. 1). CD-1 mice were responsive to all concentrations of DPX-rPA tested; however, titers from mice in the 0.05 µg dosing group did not significantly increase above the Week 3 antibody level. Notably, serum anti-rPA IgG levels were markedly higher in mice vaccinated with DPX-rPA (4 and 10 µg) compared with mice given alum-rPA (*p* < 0.001). The other concentrations evaluated did not achieve significance over alum-rPA-induced IgG levels. These findings demonstrate that rPA formulated with DPX generates quantifiable levels of circulating anti-PA antibodies in CD-1 mice as early as 3 weeks after immunization that persist for at least 20 weeks.

### Vaccination with DPX-rPA generates rapid, functional antibody response in rabbits

We next evaluated the immunogenicity of a single inoculation with DPX-rPA compared with alum-rPA in rabbits. The New Zealand White rabbit was chosen because it is well characterized as a model for inhalational anthrax.^17^ In this initial study, we used polyinosinic:polycytidylic acid (poly I:C) as an adjuvant as we have demonstrated that it can generate robust antibody responses when formulated in DPX.^18^ Based on the dose finding experiment in mice (Fig. 1), rabbits were inoculated intramuscularly with a single dose of DPX-rPA (10 µg; Week 0) or three doses of alum-rPA (10 µg; Weeks 0, 4, and 16). Serum IgG against PA were measured every 4 weeks from Week 4 until Week 36 following vaccine delivery.

**Fig. 2:**
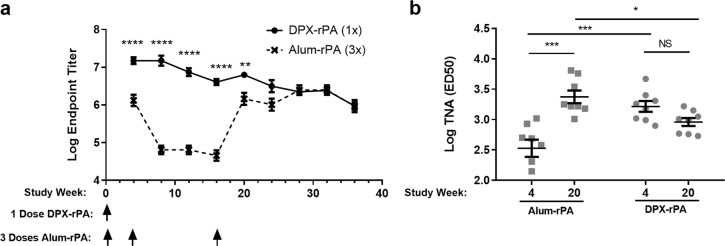
Vaccination with DPX-rPA generates rapid, functional antibody response in rabbits. **a**, **b** Groups of eight New Zealand White rabbits were vaccinated once (Week 0) with 10 µg DPX-rPA (black circles) or three times (Weeks 0, 4, and 16) with 10 µg alum-rPA control vaccine (black x). Results are shown as mean ± SEM. **a** Endpoint anti-rPA immunoglobulin G (IgG) titers, presented as Log_10_ values, between Weeks 4 and 36 post immunization determined by enzyme-linked immunosorbent assay (ELISA). Immunizations are temporally indicated with arrows along the *x*-axis. **b** Anthrax toxin-neutralizing activity of IgG antibodies, presented as Log_10_ toxin-neutralizing assay (TNA) (ED_50_ (50% effective dose)), at Weeks 4 and 20 post immunization with alum-rPA or DPX-rPA vaccines determined by TNA. Individual responses shown with mean value indicated with a line and ±SEM. Significance was determined by two-way analysis of variance (ANOVA) with Bonferroni post test; *p < 0.05, **p < 0.01, ***p < 0.001, ****p < 0.0001, NS: not significant

DPX-rPA generated significantly higher antibody titers over alum-rPA within 4 weeks of delivery (*p* < 0.001), and titers persisted for the 36-week duration of the study (Fig. 2a). Comparable antibody titers were not achieved with alum-rPA until after two boosting immunizations, administered at Weeks 4 and 16 after initial vaccine delivery.

To assess the functionality of the antibodies induced, sera samples taken at Weeks 4 and 20 were assayed for their ability to neutralize anthrax toxin in vitro using a toxin-neutralizing assay (TNA). In contrast to alum-rPA, which had minimal neutralizing activity after a single-dose, DPX-rPA produced a significantly greater neutralizing antibody response (ED_50_ (50% effective dose)) at Week 4 (*p* < 0.001) (Fig. 2b). Moreover, the degree of neutralizing activity was not significantly diminished at Week 20 in the DPX-rPA vaccine group, demonstrating collectively that DPX-rPA has the ability to maintain functional anti-rPA titers in rabbits after single vaccine treatment.

### Functional antibodies induced by DPX-rPA confer protection against lethal *B. anthracis* challenge in rabbits

To evaluate the protective efficacy of DPX-rPA, we immunized rabbits and challenged them with *B. anthracis* Ames strain spores via nose-only inhalation. In this study, we used the adjuvant Pam3CSK4 in the DPX-rPA formulation, as it also induces strong antibody responses,^18^ and was used clinically in another infectious disease-based project with DPX making it a more clinically attractive component.^14^ As a comparator, we utilized the commercially available AVA vaccine instead of alum-rPA. Anthrax challenge models in rabbits and NHPs have shown two doses of AVA diluted 1:4 provides toxin-neutralizing antibodies and 100% protection, comparable to human studies of AVA.^19^ As the amount of PA in AVA can range from 1.75 to 7 μg/mL (based on Product Specifications), at a 1:4 dilution the range of PA dose administered per 0.5 mL volume of AVA is 0.22–0.88 μg. A range of DPX-rPA doses were tested (9, 3, 1, 0.33, and 0.11) to encompass the calculated AVA PA doses and evaluate the benefit of a single administration regimen at a range of antigen doses. Rabbits were vaccinated once (Day 0) with DPX-rPA formulations or twice with AVA (Day 0, 28) and challenged on Day 70. Serum was collected throughout to monitor development of neutralizing antibody titers. All animals were terminated on Day 84.

**Fig. 3:**
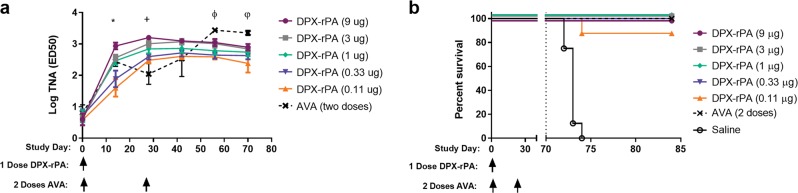
DPX-rPA confers protection against lethal *Bacillus anthracis* challenge in rabbits. **a**, **b** New Zealand White rabbits were vaccinated intramuscularly once (Day 0) with DPX-rPA (*n* = 8 per dose group) dosed at either 9 µg (purple circles), 3 µg (gray squares), 1 µg (green diamonds), 0.33 µg (dark purple downwards triangles), 0.11 µg (orange upwards triangles), or twice (Days 0 and 28) with AVA (black x, dashed line; *n* = 6). Immunizations are temporally indicated with arrows along the *x*-axis. **a** Anthrax toxin-neutralizing activity of immunoglobulin G (IgG) antibodies prior to immunization and at Days 14, 28, 42, 56, and 70 post immunization determined by TNA and shown as Log_10_ values (ED50 (50% effective dose)). Individual values below the lower limit of quantification (LLOQ) were replaced with the 1/10th of the LLOQ value (2.582) for statistical analysis purposes. Results are shown as mean ± SEM. Significance was determined by two-way analysis of variance (ANOVA) with Tukey’s multiple comparisons test, and statistics shown for relevant comparisons where at least *p* < 0.05 was detected: *, between DPX-rPA (9 μg) and DPX-rPA (0.33, 0.11 μg); +, between DPX-rPA (9 μg) and DPX-rPA (0.11 μg), between AVA and DPX-rPA (9, 3, 1 μg); ϕ, between AVA and DPX-rPA (0.33, 0.11 μg); φ, between AVA and DPX-rPA (0.11 μg). **b** Kaplan--Meier curve showing percent rabbit survival over time after inhalational challenge with *B. anthracis* spores on Day 70 (indicated with dotted line) post-DPX-rPA or post-AVA immunization. Significance was determined by Mantel–Cox test. In **b**, all DPX-rPA dosing groups and AVA were significantly compared to saline, at least *p* < 0.05

Prior to anthrax challenge, TNA (ED_50_(50% effective dose)) analyses revealed a dose-dependent response to DPX-rPA (Fig. 3a). The AVA control also induced high titer-neutralizing antibodies in rabbits, but only after administration of a second vaccination. By the time of challenge, Day 70, animals treated with two doses of AVA had the highest TNA response, but this level of toxin-neutralizing antibody was only significantly different to that in animals treated with DPX-rPA at the lowest dose, 0.11 μg. All AVA-vaccinated animals subsequently survived lethal inhalational anthrax exposure (Fig. 3b). A single dose of DPX-rPA was also 100% effective at protecting rabbits in all doses except in the lowest dose group (0.11 µg); seven of eight (87.5%) of these rabbits survived until study end. Conversely, all animals in the saline control group were either euthanized due to moribund condition or found dead within 4 days of lethal anthrax challenge. Gross necropsy and microscopic analyses revealed lesions consistent with inhalational anthrax in all saline-treated animals and in the one non-surviving rabbit from the 0.11 µg DPX-rPA dosing group. Rabbits that died tested positive for anthrax in the lung and spleen via colony formation microbial assessment. Rabbits receiving saline also tested positive for anthrax in the liver (8/8, 100%) and brain (7/8, 86%) and 75% (6/8) had anthrax in blood after Day 71 (24 h post challenge) and Day 72 (48 h post challenge); all rabbits in this group demonstrated bacteremia at least twice between Day 71 and prior to death. All surviving vaccinated animals were negative for bacteremia from Day 75 through the end of the study and none of the animals had detectable bacteria in tissues evaluated. Animals vaccinated with 3 or 9 μg rPA and those administered AVA did not demonstrate bacteremia at any time after challenge. Two animals within the 1 μg group, and one animal each in the 0.33 and 0.11 μg dose groups became bacteremic once after challenge between Days 71 and 74. While the bacteremic animal from the 0.11 μg dose group succumbed to disease, the other bacteremic animals went on to survive challenge.

### Toxin-neutralizing antibodies are generated after single immunization with DPX-rPA in non-human primates

Having established immunogenicity and efficacy in a rabbit model, we proceeded to test DPX-rPA in cynomolgus macaques, a model species that closely recapitulates human anthrax disease.^17^ We first assessed development of functional antibodies by TNA as well as vaccine safety in non-human pimates (NHPs) vaccinated once (Study Day 0) or twice (Study Days 0 and 56) with DPX-rPA (5 µg) as compared with AVA (diluted 1:4) administered twice (Study Days 0 and 28). For safety, macaques were monitored for gross and histological signs related to DPX delivery at the injection site as well as clinical observations and body weight and temperature.

**Fig. 4:**
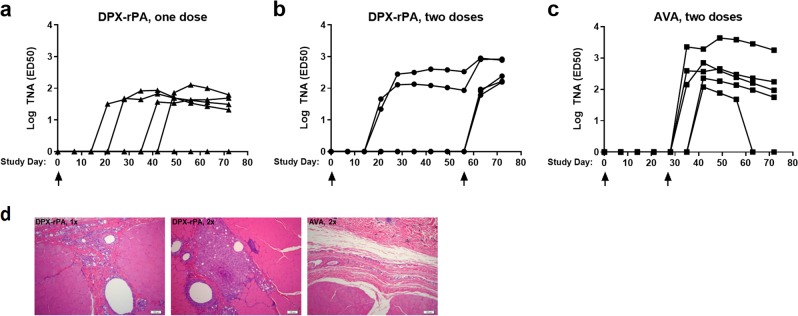
Anthrax toxin-neutralizing antibodies (TNAs) are generated after single immunization with DPX-rPA in non-human primates. **a**–**c** Groups of five cynomolgus macaques were immunized (**a**) once (Day 0; black triangles) or (**b**) twice (Days 0 and 56) with 5 µg DPX-rPA (black circles) or (**c**) twice (Days 0 and 28) with AVA (black squares). Immunizations are temporally indicated with arrows along the *x*-axis. Anthrax toxin-neutralizing activity of immunoglobulin G (IgG) antibodies, presented as Log_10_ TNA (ED_50_ (50% effective dose)), determined by TNA for each animal starting at Study Day 0 and continuing once per week until Study Day 72. Individual responses are shown, symbols indicate time points tested. **d** Representative histology of macaque hip/thigh stained with hematoxylin and eosin showing injection site after one dose of DPX-rPA, two doses of DPX-rPA, or two doses of AVA. Image magnification = ×100

Consistent with our findings in mice and rabbits, the DPX-rPA vaccine was capable of inducing high titers of toxin-neutralizing antibodies in NHPs after single-dose administration (Fig. 4a, b). Interestingly, there was wide variance across animals in initial TNA (ED_50_ (50% effective dose)) response; responses were detected in four out of five macaques in the single-dose DPX-rPA group between 21 and 49 days post injection (Fig. 4a). In the group that received two doses of DPX-rPA, two out of five generated responses by Day 21 and the remaining three all had responses by Day 63, 7 days after the second injection (Fig. 4b). In contrast, neutralizing antibody activity was only detectable in all five AVA-vaccinated macaques after a Day 28 boosting injection (Fig. 4c). Microscopic examination of the injection site collected at study termination indicated minimal to mild injection site lesions in the DPX-rPA group, probably related to either an immune response and/or site irritation and itching. Histological examination at Day 72 necropsy revealed minimal to mild DPX-rPA- or AVA-related injection site lesions with evidence of granulomatous or histiocytic inflammation (Fig. 4d). Additional lesions observed were considered to have been secondary to inflammation or to mechanical trauma of the injection. There were no abnormal clinical observations, and only minor fluctuations in body temperature were noted with individual animals. Body weight profile steadily rose from Day 7 to Day 63, but interestingly declined on Day 72 in a majority of animals. Overall, these results suggest that DPX-rPA is well tolerated, and a single dose of DPX-rPA may be sufficient to impart protective immunity.

### Single immunization with DPX-rPA protects NHPs from aerosolized challenge with lethal *B. anthracis* spores

We evaluated the ability of a single dose of DPX-rPA to protect NHPs from inhalational *B. anthracis* challenge. To mitigate the variability of the TNA response generated by the 5 µg DPX-rPA dose, separate cohorts of cynomolgus macaques were vaccinated with DPX-rPA delivered once at doses of 10 or 25 µg on Study Day 0 or twice with AVA (1:4 dilution in saline) on Study Days 0 and 28 (*n* = 6 per group). Sera were harvested on Days 0, 10, and 14 and then every 2 weeks until Day 70 when they were challenged with *B. anthracis* Ames spores.

Local vaccine reactogenicity was grossly visible in two animals, one each from the DPX-rPA (25 µg) and AVA groups, with these observations limited to very slight or barely perceptible edema or erythema. Aside from one animal who received DPX-rPA (25 µg) and exhibited moderate histological lesions (inflammation and fibrosis), DPX-rPA and AVA were well tolerated. Clinical observations during the pre-challenge period were primarily normal with only a few instances of gastrointestinal issues, including soft stool and diarrhea. No clinically significant changes were observed in body weights or temperatures recorded daily for 7 days after each vaccination.

**Fig. 5:**
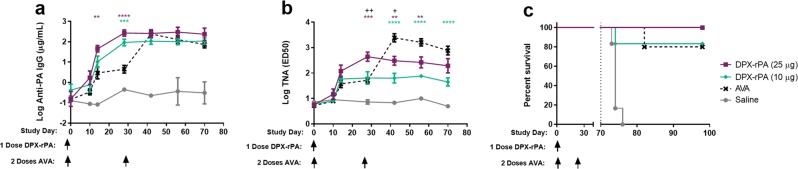
Single immunization with DPX-rPA protects non-human primates from aerosolized challenge with lethal *Bacillus anthracis* spores. **a**–**c** Groups of six cynomolgus macaques were vaccinated once (Day 0) with 10 µg (purple squares) or 25 µg (green triangles) DPX-rPA or twice (Days 0 and 28) with AVA (black x) or administered saline (gray circles) as a negative control. Immunizations are temporally indicated with arrows along the *x*-axis. **a** Anti-rPA immunoglobulin G (IgG) titers (µg/mL), presented as Log 10 values, on Days 0, 10, and 14 and then every 2 weeks until Day 70 post immunization determined by enzyme-linked immunosorbent assay (ELISA). **b** Anthrax toxin-neutralizing activity (TNA) of IgG antibodies, presented as Log_10_ TNA (ED_50_ (50% effective dose)), at Days 0, 10, and 14 and then every 2 weeks until Day 70 post immunization determined by TNA. Significance for **a**, **b** was determined by two-way analysis of variance (ANOVA) with Tukey’s multiple comparisons test, and results are shown as mean ± SEM. In purple (*) shows comparison between 25 μg DPX-rPA vs. alum-rPA, in green (*) shows 10 μg DPX-rPA vs. alum-rPA, and in black (+) shows comparison between 25 μg DPX-rPA and 10 μg DPX-rPA; +*p* < 0.05, **or ++*p* < 0.01, ***p < 0.001, ****p < 0.0001. **c** Kaplan–Meier curve showing percent macaque survival over time after inhalational challenge with *B. anthracis* spores on Day 70 (indicated with dotted line) post-DPX-rPA or post-AVA immunization. Significance was determined by Mantel–Cox test and is shown comparing each group to saline control, **p < 0.001, ***p < 0.0001. No statistical significance was detected between the three vaccinated groups

A single administration of DPX-rPA, regardless of dose, yielded high titers of anti-rPA IgG in NHPs by Day 14 and that peaked at Day 70. Relative to saline, all three vaccinated groups generated anti-rPA IgG titers by Day 14, which were maintained until the end of study (Fig. 5a). There was no statistical difference between the anti-rPA titers generated by DPX-rPA at either dose level, and both DPX groups generated statistically higher titers by Day 28 when the AVA groups were vaccinated with their second dose. In general, TNA (ED_50_) kinetics paralleled the anti-rPA IgG response in both DPX-rPA groups (Fig. 5b). AVA-vaccinated macaques developed a similarly robust serum IgG and TNA response, particularly after the Day 28 boosting injection, and that peaked 2 weeks later on Day 42. Anti-rPA antibody responses were not detected in macaques vaccinated with saline alone.

DPX-rPA (25 µg) was 100% effective at protecting macaques from lethal challenge with *B. anthracis* spores (Fig. 5c). One animal from each of the DPX-rPA (10 µg) and AVA groups died after anthrax inhalation before study completion (Day 98). All saline control animals died within 6 days of anthrax challenge. Examination of tissue and blood from animals that succumbed to disease revealed elevated serum C-reactive protein, positive terminal bacteremia, gross necropsy lesions, and histologic lesions consistent with fulminant anthrax infection. In addition, abnormal clinical observations, hematologic changes, and body temperature increases or decreases occurred more frequently in saline control animals than in the other groups. Animals that survived had active or resolving inflammation, consistent with lesions typically present after *B. anthracis* exposure. The incidence of bacteremia in surviving animals and animals that died prior to study completion is presented in Table 1. Interestingly, three of six macaques given DPX-rPA (25 µg) tested positive for bacteremia at Day 72, 2 days post challenge; bacteremia resolved by Day 75 through the end of study. All six saline control animals were bacteremic at one or more time points after infection, beginning on Day 72, which was associated with early termination. Each of the animals from the DPX-rPA and AVA groups that died prior to study completion were bacteremic on at least one-day post-challenge and each animal had anthrax-positive terminal samples. Furthermore, each of these animals had lower TNA and anti-rPA IgG levels than the mean of their groups at Day 70. No surviving animals from these groups had positive bacteremia tests. These results suggest that single-dose delivery of DPX-rPA is equally as effective at protecting against aerosolized anthrax infection as two doses of commercial AVA.

**Table 1:** Incidence of bacteremia post *B. anthracis* challenge

Group	No. of animals	No. of survivors	Non-survivors^a^	Survivors^a^
DPX-rPA (25 µg)	6	6	0/0	3/6^b^
DPX-rPA (10 µg)	6	5	1/1	0/5
AVA	5	4	1/1	0/4
Saline	6	0	6/6	0/0

*AVA* anthrax vaccine adsorbed, *rPA* recombinant protective antigen

^a^No. with bacteremia/no. of animals

^b^All three tested positive on Day 72, but were negative by Day 75

## Discussion

Herein, we have demonstrated that a single-dose delivery of DPX-rPA provides rapid induction of toxin-neutralizing antibodies compared to an alum formulation, and comparable immune protection from aerosolized anthrax infection in rabbits and NHPs to two-dose delivery of the currently licensed anthrax vaccine, AVA. DPX is a highly versatile, immunogenic, water-free, lipid-in-oil formulation that preliminary clinical results suggest can be safe and effective in both infectious disease and cancer settings.^14–16^ Subunit vaccine approaches have commonly relied on the alum formulation to facilitate immune responses to purified PA.^5,8^ The DPX platform utilizes lipids to incorporate diverse antigens and adjuvants into an oil-based formulation without an emulsion. This unique property holds antigens at the site of immunization and facilitates active antigen uptake,^11,20^ resulting in robust immune responses. By incorporating different adjuvants, the immune responses can be tailored for specific applications. In this study, poly I:C (TLR3 agonist) and Pam3CSK4 (TLR1/2) were both tested in rabbits. Although the antibody titers obtained with the poly I:C formulation were higher, the titers obtained by the Pam3CSK4 were still effective and this adjuvant has been used in DPX-RSV, which induced strong antibody titers in clinical evaluation.^14^

Administration of DPX-rPA rapidly generated high titers of anti-rPA IgG after single injection in all species tested. Moreover, DPX-rPA antibodies had consistently greater anthrax toxin-neutralizing activity compared to AVA antibodies generated after a single injection. In NHPs, anti-rPA IgG and TNA responses were maintained during the pre-challenge period with nominal variability. AVA, by comparison, induced an equivalent immune response only after two doses. An advantage of a subunit vaccine approach is the ability to more accurately tailor antigen doses. When DPX-rPA was tested with comparable PA doses to that in AVA, 0.11–0.33 μg (Fig. 3), it generated comparable TNA results and protection in challenge, even though animals received a single vaccination compared to two doses of AVA. Further optimization of the formulation demonstrated that higher TNA results can be achieved with increasing rPA dose while maintaining an acceptable safety profile. Comparable titers could not be achieved using alum adjuvant for rPA (Figs. 1, 2), demonstrating that the unique properties of the DPX platform could account for the potent response. The rapid and sustained response elicited by single DPX-rPA delivery relative to two dose AVA suggests DPX-rPA has the potential to be an effective as a pre-exposure as well as post-exposure vaccine option, although the latter remains untested to date. Regarding post-exposure treatment, the current approved regimen is a series of three AVA injections over 1 month in conjunction with antibiotics, which the CDC recommends be administered for five to six consecutive weeks. Concerns regarding antibiotic resistance, the feasibility of administering antibiotics to a large population during an anthrax attack, and the risk that residual spores may germinate after antimicrobial treatment are all limitations of this approach. Proof-of-concept studies in rabbits have shown that vaccines that produce a rapid anti-PA IgG response, as demonstrated with DPX-rPA, and when co-administered with short-term antibiotic treatment have the potential to minimize duration of antibiotic use, but still provide complete protection.^21^

We found that 100% of rabbits receiving a single dose of DPX-rPA as low as 0.33 µg survived anthrax challenge, despite having a mean 2-log fold less TNA response than AVA-treated rabbits prior to challenge. TNA responses are typically described to be correlative of protection in this model, and although they have waned in the DPX groups, they are still detectable. By time of challenge, 70 days post immunization, it is likely that mature memory B cell responses have developed and the level of antibodies is still sufficient for protection.^22^ In this study, we did not perform post-challenge test for anti-PA, which may have helped to clarify this theory, it will be considered in future studies, as could the incorporation of other immune measures such as T cell responses. At an antigen dose as low as 0.11 µg, DPX-rPA provided statistically significant protection from inhalational anthrax when compared to unvaccinated controls. Interestingly, in contrast to the results observed in rabbits, we observed a trend towards a positive association between vaccine concentration, TNA response, and survival outcome in NHPs. The highest dose of DPX-rPA evaluated (25 µg) elicited the strongest TNA response and conferred complete anthrax protection, whereas a dose of 10 µg DPX-rPA was associated with 83% post-challenge survival. Immunization with AVA was associated with 80% post-challenge survival. Despite these dichotomous findings, numerous studies across animal models have shown that TNA titers are a reliable predictor of surviving anthrax infection.^23^ Our data imply that DPX-rPA-induced immunity may be more efficient in rabbits than in NHPs, which should be an important consideration when utilizing DPX technology to bridge animal efficacy and human immunogenicity data to predict efficacy in humans.

Because this study may be used to inform decisions regarding future work, it is important to note animals that had survival outcomes that differed from the majority of the animals in their groups. One AVA-vaccinated NHP that succumbed to *B. anthracis* infection 12 days post challenge had anti-rPA IgG results similar to the rest of the group, but lower TNA levels at Day 70. Gross findings at necropsy were limited to red discoloration of the meninges, affecting the entire brain, with only minimal inflammation in kidney, lung, lymph nodes, and spleen. One theory is that, despite high levels of functional anti-rPA IgG in peripheral circulation, the blood–brain barrier became compromised by circulating toxin early post challenge, resulting in entrapment of *B. anthracis* bacteria in the brain and meningitis. These results suggest that IgG titer may not always correlate with survival.

Visible vaccination site reactions were minimal in all NHPs evaluated, with a single animal in the DPX-rPA (25 µg) and AVA groups having very slight edema or erythema at the vaccination site on Day 1 or 2 following the first vaccination. Histological evidence of reactogenicity (inflammation and/or fibrosis) was present in 83% (5/6) of animals receiving 25 µg DPX-rPA and 60% (3/5) of animals receiving 10 µg DPX-rPA, compared with 50% of animals receiving AVA (3/6) and 67% (4/6) of animals receiving saline. Although AVA vaccination is considered safe, with mild injection site reactions most commonly reported, the potential development of adverse events increases with the multiple doses required.^5,6^ Subcutaneous delivery of 0.25–0.1 mL DPX in humans, when formulated with cancer immunogen survivin, causes minimal to mild induration and erythema at the injection site in a majority of patients.^16^ This same delivery format was recently used for testing DPX-RSV, a respiratory syncytial formulation, in humans. In this study, two 0.05 mL doses of DPX-RSV administered to healthy adults (50–64 years) 56 days apart resulted in robust antigen-specific antibodies, which were sustained for at least 1 year and little evidence of injection site reactogenicity.^14^

Our study did not evaluate the duration of survival with DPX-rPA beyond 2 weeks after challenge in rabbits and 4 weeks in NHPs. Data in rhesus macaques suggest that three intramuscular injections of AVA may confer protection from lethal anthrax for up to 4 years.^24^ In human survivors of inhalational anthrax, anti-PA IgG were identified 8 to 16 months after onset of clinical symptoms. In the same study, peak anti-PA IgG levels correlated strongly with PA-specific IgG memory B cell frequency, which persisted in patient sera for at least 1 year after infection.^25^ Future studies aim to assess the long-term protective immunity of DPX-rPA against inhalational anthrax exposure.

This study demonstrates that DPX-rPA generates neutralizing antibody titers towards anthrax PA toxin in multiple animal models. After only a single injection, DPX-rPA increased survival in rabbits and NHP challenged with *B. anthracis*. Overall, our findings demonstrate that DPX-rPA is well tolerated and may offer an improved efficacy profile in comparison to AVA, warranting further study.

## Methods

### Animal models

All procedures were conducted in accordance to guidelines set by the Canadian Council on Animal Care or American Association for Laboratory Animal Sciences, and studies were approved by the institutional animal care ethics committees where the studies were conducted (Battelle Institutional Animal Care and Use Committee, Southern Research Institutional Animal Care and Use Committee, Dalhousie University Committee on Laboratory Animals). For the immunogenicity studies, CD-1 mice (female, aged 6 to 8 weeks) and New Zealand White rabbits (female, 2–3 kg) used in Figs. 1 and 2, respectively, were obtained from Charles River Laboratories (St. Constant, Quebec) and experiments were performed at IMV Inc. (Halifax, Canada). Mice were housed under filter-top conditions and rabbits were housed in groups of eight. All mice and rabbits were fed and watered ad libitum and housed with environmental enrichments. Male and female cynomolgus macaque NHPs used in Fig. 4, ranging from 2.6 to 8 years of age when received, were obtained from World Primates Inc. (Miami, FL, USA), University of Illinois at Chicago (Chicago, IL, USA), Covance Research Products Inc. (Alice, TX, USA), and SNBL, USA Scientific Resource Center (Alice, TX, USA). NHP experiments were performed at Southern Research Institute (Birmingham, AL, USA). NHPs were individually housed during pre-study and study periods per requirements set forth in the Animal Welfare Act and Guide for the Care and Use of Laboratory Animals.

For the challenge studies, New Zealand White rabbits (male and female, aged approximately 13 weeks when used) used in Fig. 3 were obtained from Charles River and experiments were performed at Southern Research Institute. Rabbits were single-housed in primary standard cages in an ABSL1/2 room. Seven to eight days prior to anthrax challenge, rabbits were transferred and acclimated to an ABSL-3 facility. Male and female cynomolgus macaque NHPs used in Fig. 5, ranging from 2.6 to 4.1 years of age at randomization, were procured from Covance Research Products (Alice, TX, USA) and experiments were performed at Battelle Biomedical Research Center (Columbus, OH, USA). NHPs were verified negative for Simian immunodeficiency virus, Simian T-lymphotrophic Virus-1, tuberculosis, *Macacine herpesvirus* 1 (herpes B virus), Simian retroviruses 1 and 2, and *Trypanosoma cruzi*. NHPs were transferred from a BSL1/2 facility and housed individually in a BSL3 facility 8 to 10 days prior to challenge and 28 days after challenge.

All animals used for immunogenicity and challenge studies were housed in environmentally monitored, well-ventilated rooms and screened for pre-existing antibodies to PA by anti-PA IgG ELISA.

### Preparation of recombinant PA

rPA was obtained from DRDC and produced in *Bacillus megaterium*, List Biologicals (Campbell, CA, USA) and produced in modified *B. anthracis*, or Pfenex produced in *Pseudomonas fluorescens*.

### Vaccine preparation and immunization

DPX-rPA and alum-rPA were provided by IMV Inc. (Halifax, Canada), but AVA was provided by the CDC. Test and vehicle/control article formulations were prepared fresh on the day of dosing in accordance with the method provided. DPX-rPA was prepared as previously described^19^ containing a poly I:C (Thermo Fisher) or Pam3CSK4 (EMC Microcollections) adjuvant and a 10:1 mixture of DOPC (1,2-dioleoyl-*sn*-glycero-3-phosphocholine):cholesterol (Lipoid GmBH, Germany) or S100:cholesterol (Lipoid GmBH), see Supplementary Table 1 for complete formulation details for each figure. The source of rPA was DRDC, List Biologicals, or Pfenex Inc.^26^ To reconstitute DPX-rPA, Montanide ISA 51 VG (Seppic, France) was added to the lyophilized DPX-rPA product (consisting of rPA, lipids, and adjuvant). For studies testing a series of rPA antigen concentrations, the prepared DPX-rPA was then diluted with “empty vaccine” (lipids/adjuvant in oil) to obtain the desired concentration of antigen. Alum-rPA formulation was prepared by diluting rPA antigen stock in sodium phosphate buffer (0.1 M, pH 6.0), followed by the addition of Adju-Phos^®^ (Brenntag) and mixed well by gentle shaking for 5 min. AVA was diluted with sterile saline 1:4 for administration. Two different batches of AVA were used throughout the studies: Lot FAV392A (Figs. 3 and 5) FAV306 (Fig. 4).

All vaccine inoculations were administered via intramuscular injection. Mice received a total dose volume of 0.05 mL DPX-rPA or alum-rPA, split into two equal portions delivered to the left and right caudal thigh. Rabbits received 0.1 mL of DPX-rPA or 0.5 mL of AVA administered in the left or right hind limb. NHPs received a dose of 0.1 mL of DPX-rPA or 0.5 mL of AVA administered in the left or right hip/thigh. In the case where two doses were given, the second injection was administered on the alternate thigh or limb. After dosing, the perimeter of the dose site was marked with indelible ink and monitored for injection site reactions.

### Inhalation challenge

New Zealand White rabbits for inhalation challenge experiments were transferred and acclimated to the ABSL-3 facility 7–8 days prior to the challenge. On Day 70, rabbits were sedated with acepromazine (0.25–5.0 mg/kg, intramuscularly (i.m.)) and challenged using a custom-designed nose-only inhalation mask placed over the muzzle that administered a target dose of ±200 LD_50_ (2.2 × 10^7^ colony-forming unit (CFU)) in a cumulative inhaled volume of 24.0 L using real-time plethysmography. The average actual challenge dose for rabbits was 211 LD_50_. The inhalation exposure time ranged from 10.5 to 20.2 min. Rabbits were observed twice daily for signs of mortality and moribundity days 0–70 and three times daily thereafter. Body weights were taken daily throughout the duration of the study. Temperatures were collected daily on days 0–70, three times a day on Days 71–77, and twice daily on Days 78–84 using implanted micro-identification devices. Blood was taken from the central ear artery and was either processed for serum or plated on agar plates for bacteremia assessment daily between Days 70 and 84.

Non-human primates for inhalation challenge experiments were transferred into the BSL3 facility 8–10 days prior to challenge to allow time for acclimation. The animals were challenged via inhalation with *B. anthracis* Ames spores on Day 70 as previously described.^4,27^ Briefly, the animals were anesthetized with Telazol (3 mg/kg, i.m.) and placed into a plethysmography chamber and a Class III Biosafety Cabinet (BSCIII) system and were aerosol challenged by head-only inhalation of *B. anthracis* Ames spores aerosolized by a Collison nebulizer with a targeted ±200 LD_50_ (1.24 × 10^7^ CFU) dose. The average actual challenge dose for NHPs was 283 LD_50_. The aerosol challenge duration was based upon a calculated starting concentration and a cumulative minute volume gathered real time throughout the exposure. Following exposure, the head of the animal was decontaminated. NHPs were observed twice daily for mortality and moribundity. Once daily, animals had temperatures collected via implantable micro-identification device. Blood was collected for hematology, C-reactive protein, and qualitative bacteremia on Days 2, 5, 8, 12, 14, and 28 post challenge and at the time of death or euthanasia, if possible, into tubes containing EDTA. Animals were weighed at the time of challenge, and at either 28 days post challenge (survivors) or the time of death or euthanasia.

### Anti-PA IgG ELISA

For mice and rabbit experiments, ELISA plates were coated overnight with recombinant PA antigen (BEI Resources, Defense Research and Development Canada) and then plates were washed, blocked, and washed again. Controls or archived serum samples from mice or rabbits were added, incubated, and subsequently washed. Alkaline phosphatase-linked protein G (mice) or A (rabbits) with activity to the appropriate animal IgM and IgG was added and subsequently incubated and washed. Optical density at 405 nm for alkaline phosphatase measurement was read on a plate reader. Endpoint titers, expressed in Log_10_, were defined as the reciprocal of the highest dilution above the cutoff value determined from the control serum; cutoff values were calculated using a 95% confidence interval.^28^

The anti-rPA IgG ELISA analysis in NHPs was conducted according to validated procedures at Batelle Biomedical Research Center as previously described.^27^ Briefly, the assay is designed to quantify IgG antibodies against anthrax PA using purified rPA as the solid-phase immobilized antigen and an enzyme-conjugated anti-γ chain secondary antibody as the reporter system. The assay is reported as the Log_10_ serum mean concentration of anti-PA-specific IgG (μg/mL).

### Toxin neutralization assay

Serum from rabbits and NHP was collected in BSL3 and passed through 0.22 µM filters and plated on blood agar to confirm sterility before use. Sera were prepared from blood samples and stored at <–70 °C until use for TNA.

Methods for the TNA in rabbits and NHPs have been previously described and conducted according to optimized standard operating procedures.^27^ Briefly, J774A.1 mouse macrophage cells were seeded at between 2.0 × 10^4^ and 5.0 × 10^4^ cells per well on Day 1 and allowed to incubate overnight at 35–38 °C in a CO_2_ incubator. On Day 2, lethal toxin (LT), composed of rPA and recombinant LF (rLF), was prepared in 2X complete media and diluted in each well to reach the final concentrations in the assay. Dose of rPA and rLF was determined by titration as the dose required for 50% lysis using a reference serum and ranged between 100–300 and 50–250 ng/mL, respectively. LT was incubated with 2-fold serial dilutions of serum (starting at a 1:50 dilution) and then added to J774A.1 cells, followed by an approximately 4-h intoxication period in a CO_2_ incubator at 35–38 °C. MTT (3-(4,5-dimethyl-2-thiazolyl)-2,5-diphenyl-2*H*-tetrazolium bromide) was added and cells were placed in a CO_2_ incubator at 35–38 °C for approximately 2 h, lysed with solubilization solution, sealed, and then incubated overnight at 35–38 °C. The optical density values for each plate were read on a microplate reader at a wavelength of 570 nm or 590 nm using a 690 nm reference wavelength. The ED_50_ was defined as the reciprocal of the dilution of a serum samples that results in 50% neutralization of the LT, as represented by the inflection point of the sigmoidal curve generated by the serial dilution of the serum. For Figs. 3 and 4, the ED_50_ was calculated by an iterative curve-fitting algorithm using the SoftMax Pro software (version 4.3.1). For Fig. 5, the ED_50_ was calculated using the TNA SAS^®^ program. The ED_50_ was defined as the reciprocal of the dilution of a serum sample that results in 50% neutralization of the LT, as represented by the inflection point of the sigmoidal curve generated by the serial dilution of the serum. For Figs. 3 and 4, the ED_50_ was calculated by an iterative curve-fitting algorithm using the SoftMax Pro software (version 4.3.1). For Fig. 5, the ED_50_ was calculated using the TNA SAS^®^ program.

### Histology

Tissues were collected from animal at necropsy on study termination. Fixed tissues of hip/thigh from cynomolgus macaques were trimmed, processed, and microtomed (approximately 5 µm sections). The tissue sections were mounted on glass slides, stained with hematoxylin and eosin, and coverslipped. All slides of all animals in all groups were submitted to a veterinary pathologist for histopathologic evaluation. Each lesion was listed and coded for the most specific topographic and morphologic diagnoses, severity, and distribution. A four-step grading system of minimal, mild, moderate, or marked was used to rank the severity of microscopic lesions for comparison among groups.

### Hematology, C-reactive protein, and bacteremia

Hematology was evaluated for the following parameters: white blood cell count, differential leukocyte count, neutrophil/lymphocyte ratio, hemoglobin, hematocrit, red blood cell count, mean corpuscular volume, mean corpuscular hemoglobin, mean corpuscular hemoglobin concentration, red cell distribution width, platelet count, and platelet volume. The Advia^®^ 120 Hematology System was used for these evaluations. C-reactive protein levels were assessed using the Siemens Advia^®^ 1200 Chemistry Analyzer.

Qualitative bacteremia assay was performed by streaking 40–100 μL of whole blood onto blood agar plates using a sterile inoculating loop. Plates were incubated at 37 °C between 48 and 78 h. Samples that resulted in any colonies consistent with *B. anthracis* morphology (γ-hemolytic, white colonies, 4–10 mm in diameter with a rough appearance and irregular edges) following incubation on blood agar were documented as positive. Results were documented as negative when *B. anthracis* colonies were not present after incubation.

### Reporting summary

Further information on experimental design is available in the Nature Research Reporting Summary linked to this article.

## Supplementary information

Suppl Table 1

Reporting Summary

## Data Availability

The data that support the findings of this study are available from the corresponding author upon reasonable request.

## Supplementary information

**Supplementary information** accompanies the paper on the *npj Vaccines* website (10.1038/s41541-019-0102-z).
