# Supplementary material for: Single dose of DPX-rPA, an enhanced-delivery anthrax vaccine formulation, protects against a lethal Bacillusanthracis spore inhalation challenge
Source: NPJ Vaccines. 2019 Feb 8;4:6. doi: 10.1038/s41541-019-0102-z (PMC6368554; doi:10.1038/s41541-019-0102-z)
Supplement: Supplementary file 1 — Suppl Table 1 [file 41541_2019_102_MOESM1_ESM.docx]

**Supplementary Table 1: DPX formulation details for each figure.**

| Fig | Dose Volume | rPA/ Dose | rPA Source | Adjuvant/ Dose | DPX Lipids |
| --- | --- | --- | --- | --- | --- |
| 1 | 0.05 mL | 0.05, 0.5, 2, 4, 10 ug | Pfenex | Pam3CSK4 (2 ug) | DOPC:cholesterol (132 mg/mL) |
| 2 | 0.2 mL | 10 ug | DRDC | Poly I:C (50 ug) | DOPC:cholesterol (132 mg/mL) |
| 3 | 0.1 mL | 0.11, 0.33, 1, 3, 9 ug | Pfenex | Pam3CSK4 (4 ug) | DOPC:cholesterol (132 mg/mL) |
| 4 | 0.1 mL | 5 ug | List Biologicals | Pam3CSK4 (2 ug) | S100:cholesterol (132 mg/mL) |
| 5 | 0.1 mL | 10, 25 ug | Pfenex | Pam3CSK4 (4 ug) | DOPC:cholesterol (132 mg/mL) |
